# Analysis and Expansion of the Role of the *Escherichia coli* Protein ProQ

**DOI:** 10.1371/journal.pone.0079656

**Published:** 2013-10-25

**Authors:** Daniel T. Sheidy, Ryszard A. Zielke

**Affiliations:** 1 Department of Molecular, Cellular, and Developmental Biology, University of Michigan, Ann Arbor, Michigan, United States of America; 2 Department of Pharmaceutical Sciences, Oregon State University, Corvallis, Oregon, United States of America; New England BioLabs, United States of America

## Abstract

The decrease in proline transport by the proline porter ProP in a Δ*proQ* strain has been well documented; however, the reason for this phenotype remains undefined. Previous studies have speculated that ProQ facilitates translation of *proP* mRNA. Here, we demonstrate that ProQ is enriched in the polysome fractions of sucrose gradient separations of *E. coli* lysates and the 30S fractions of lysates separated under conditions causing ribosomal subunit dissociation. Thus, ProQ is a *bona fide* ribosome associated protein. Analysis of *proQ* constructs lacking predicted structural domains implicates the N-terminal domain in ribosome association. Association with the ribosome appears to be mediated by an interaction with the mRNA being translated, as limited treatment of lysates with Micrococcal Nuclease maintains ribosome integrity but disrupts ProQ localization with polysomes. ProQ also fails to robustly bind to mRNA-free 70S ribosomes *in vitro*. Interestingly, deletion of *proP* does not disrupt the localization of ProQ with translating ribosomes, and deletion of *proP* in combination with the *proU* operon has no effect on ProQ localization. We also demonstrate that ProQ is necessary for robust biofilm formation, and this phenotype is independent of ProP. Binding studies were carried out using tryptophan fluorescence and *in vitro* transcribed *proP* mRNAs. *proP* is transcribed from two differentially regulated promoters, and ProQ interacts with *proP* mRNA transcribed from both promoters, as well as a control mRNA with similar affinities. In total, these data suggest that ProQ is positioned to function as a novel translational regulator, and its cellular role extends beyond its effects on proline uptake by ProP.

## Introduction

 The maintenance of osmotic balance is essential for the fitness and survival of bacteria. One mechanism used by prokaryotes to achieve this balance in hyperosmotic environments is the import of osmoprotectant molecules which balance internal and external osmolarity and prevent the flow of water out of the cell [for review see 1]. Several membrane bound transporters exist with a variety of specificities for different molecules. One such transporter, ProP, senses hyperosmotic stress, and responds by importing proline and glycine betaine [2-4]. Examination of the transcriptional regulation of *proP* has revealed a complex network of both growth-phase and osmolarity dependent control. Briefly, *proP* transcription can occur from a proximal (P2) or distal (P1) promoter [5]. Transcription from the P2 promoter occurs as cells transition from the logarithmic-growth phase into stationary phase and is dependent upon the stationary-phase sigma factor RpoS. Transcription from the P2 promoter is further enhanced by the nucleoid-associated factor Fis [6,7] and cyclic AMP receptor protein (CRP) [8]. The binding of Fis and CRP inhibit transcription from the P1 promoter [7,9]. The P1 promoter is activated after subculture into fresh media, and is responsible for responding to upshifts in media osmolarity [5,9,10]. 

 Beyond transcription, ProP activity is modulated by the cytoplasmic effector ProQ [11]. ProQ is a 232-residue protein, predicted to contain two structural domains, tethered by an unstructured linker [12,13]. The N-terminal domain has been modeled on the structure of the RNA-binding, translational regulator FinO [12,14], and the C-terminal domain has been modeled on the RNA chaperone Hfq [15]. Biochemical studies have been performed to support the structural predictions. The FinO-like domain, as well as the full length protein, are capable of binding to a model dsRNA template. The FinO-like domain also facilitates strand exchange, and both domains promote duplexing between complimentary strands of RNA [15]. Thus, ProQ behaves as an RNA chaperone. The mechanism behind ProQ regulation of ProP activity, however, remains largely unknown. Disruption of the *proQ* locus has no effect on the transcription of *proP*, but the proline uptake activity of ProP is significantly decreased in a Δ*proQ* strain [11,16]. A post-translational mechanism was initially proposed after ProP protein levels appeared unchanged in a Δ*proQ* strain [16]; however, a direct physical interaction between ProP and ProQ has not been found. Most recently it was shown that, at osmolalities lower than those previously examined, ProP levels are decreased in a *proQ* mutant [15]. Additionally, as cells enter stationary phase, there is a modest decrease in the level of ProP in a *proQ* mutant compared to wild type [15]. In light of these findings, and the homology models comparing the ProQ domains to known RNA-binding proteins, a post-transcriptional mode of regulation is likely.

 It had been reported in a high throughput study that ProQ was associated with ribosomes [17]. This led to the hypothesis that ProQ regulates ProP activity at the level of translation. In this study, we verify that ProQ is associated with ribosomes *in vivo*, and we characterize this association under various conditions. We also determine which domains of ProQ are important for association. We demonstrate that ProQ binds tightly, but non-selectively to *in vitro* transcribed *proP* mRNA, and we report the *K*_*D*_ values for P1, P2, and an mRNA whose translation is not predicted to be dependent upon ProQ. Though we demonstrate that mRNA integrity is important for the association of ProQ with translating ribosomes, disruption of the *proP* locus, as well as the closely related *proU* operon, does not affect ProQ localization in polysome profiles. It had also been reported in a high throughput study that a *proQ* mutant exhibits decreased biofilm formation. Here we verify that result by complementing the phenotype and show that a *proP* mutant strain is not defective in biofilm formation. It follows that ProQ may act as a translation factor for a broader subset of mRNAs.

## Materials and Methods

### Bacterial strains and growth conditions

*E. coli* strains used in this study are listed in Table 1. Strains were grown at 37°C in Luria-Bertani (LB) broth (10 g tryptone, 5 g yeast extract, 10 g NaCl per liter). Cultures of JM6733 containing plasmids were supplemented with 20 µg/mL chloramphenicol (BioExpress). JM6733 (Δ*proQ::KAN*), JM6753 (Δ*proP::KAN*), JM6754 (Δ*proV::KAN*), JM6755 (Δ*proW::KAN*), and JM6877 (Δ*proX::KAN*) were constructed by P1 transduction [18] of KEIO-collection mutants, JW5300, JW4072, JW2652, JW2653, and JW2654 respectively, into a clean BW25113 background [19]. JM6881 (Δ*proVWX::KAN*) was made using the λ_red_ recombination system [20]. JM6906 (Δ*proP::FRT*) was constructed by transformation of JM6753 (Δ*proP::KAN*) with pCP20, and subsequently, screening for sensitivity to kanamycin [21]. JM6926 (Δ*proP::FRT*, Δ*proVWX::KAN*) was constructed by P1 transduction of JM6906 by phage grown on JM6881. Genotypes were verified using primers flanking the genomic regions of interest (Table 2). Culture growth was monitored by measuring the absorbance at 600 nm.

**Table 1:** Strains and Plasmids.

Strain	Genotype	Origin
BW25113	∆(*araD-araB*)*567*, ∆*lacZ4787*(::rrnB-3), lambda^-^, *rph-1*, ∆(*rhaD-rhaB*)*568*, *hsdR514*	[20]
JM6733	BW25113, Δ*proQ*::KAN	[19], This study
JM6753	BW25113, Δ*proP*::KAN	[19], This study
JM6754	BW25113, Δ*proV*::KAN	[19], This study
JM6755	BW25113, Δ*proW*::KAN	[19], This study
JM6877	BW25113, Δ*pro*X:KAN	[19], This study
JM6881	BW25113, Δ*proVWX*::KAN	This study
JM6906	BW25113, Δ*proP*::FRT	This study
JM6926	BW25113, Δ*proP*::FRT, Δ*proVWX*::KAN	This study
Plasmid	Description	Origin
	pCA24N-ProQ	[23]
pDS1	pCA24N-*ProQΔ115N*	This study
pDS2	pCA24N-*ProQΔ181C*	This study
pDS3	pCA24N-*ProQΔ124-180*	This study
pDS4	pCA24N-*ProQ116-180*	This study
pDS5	pCA24N-*ProQ2-123*	This study
pDS6	pMCSG7-ProQ	[24], This study
pMR20	pMR20 empty vector	[27]
pMR20-ProQ	pMR20 containing *proQ* ORF plus 500 bp upstream	This study
pCP20	Flip Recombinase containing vector	[21]

**Table 2:** Oligonucleotide sequences.

Name	Sequence (5’ - 3’)	Function
DS001	GAT CGA GCT CTC TGA CAT TTC AGC TCT GAC T	Construction of *proQΔ124-180*
DS002	GAT CGA GCT CTT CGC GTT TTT TCG CTT GCT G	Construction of *proQΔ124-180* and *proQ1-123*
DS003	GAT CGA GCT CCC CCT ATG CGG CCG CTA A	Construction of *proQΔ181C* and *proQ1-123*
DS004	GAT CGA GCT CAA CCG GGG TGT GCT GTT C	Construction of *proQΔ181C*
DS005	GAT CGA GCT CGA ACA GCA AGC GAA AAA ACG C	Construction of *proQΔ115N* and *proQ181-232*
DS006	GAT CGA GCT CCC TCA GGG CCG GAT CCG T	Construction of *proQΔ115N* and *proQ181-232*
DS007	GAT CGG TAC CCC CCT ATG CGG CCG CTA A	Construction of *proQ116-180*
DS008	GAT CGG TAC CAA CCG GGG TGT GCT GTT C	Construction of *proQ116-180*
DS037	GAT CGA GCT CGT CAT TAA CTG CCC AAT TCA GGC GTC	pBluescript-*proPp1*
DS038	GAT CGA GCT CAG AGA TTG CAT CCT GCA ATT CCC G	pBluescript-*proPp2*
DS039	GAT CGG TAC CTT ATT CAT CAA TTC GCG GAT GTT GCT GC	pBluescript-*proP* reverse
rpoS-T7-forw-SacII	CCG CGG CCG ACA ATT ACG TAT TCT GA	pBluescript-*rpoS*
rpoS-T7-rev-PstI	CTG CAG TTG AGA CTG GCC TTT CTG AC	pBluescript-*rpoS*
DS046	TAC TTC CAA TCC AAT GCC ATG GAA AAT CAA CCT AAG TTG	Construction of 6His-TEV-ProQ
DS047	TTA TCC ACT TCC AAT GTT ATC AGA ACA CCA GGT GTT CTGC	Construction of 6His-TEV-ProQ
DS048	CGC AGG ATA ATC AAC GGA TAA CG	*proQ* genotyping
DS049	ATT TGA TCA GCA CGC GTG ATA TC	*proQ* genotyping
DS054	CAG AGA TTG CAT CCT GCA ATT CCC	*proP* genotyping
DS055	CCT GAT AAG ACA GCG TCA CAT CAG	*proP* genotyping
DS056	GCT CGC ATC AAT ATT CAT GCC ACA	*proV* genotyping
DS057	GTA CTG GTC AGC CAG TCA GCA	*proV* genotyping
DS058	TAG CGA GTT GCT CTC TCA TGT CG	*proW* genotyping
DS059	AGT TTG TGT AGA GAT AAG CGT GGC	*proW* genotyping
DS060	GGC CCT GTT GGT CTG CTG AC	*proX* genotyping
DS061	GTC GCA TCA GGC ATT GTG CAC	*proX* genotyping
DS062	GGT TTC TGG CTG CCG ATG TAT	*proVWX* (*proU*) genotyping
DS063	CAG CCT ACA CCC TGC TGC GGG TAG TGA TAT	*proVWX* (*proU*) genotyping
DS076	GAT CTC ACT AGC CAT CCT GCA AAC CTT CAC G	*proP in vitro* transcription template
DS077	ATT GGG TAA TAT ATC GAC ATA GAC AAA TAA AGG AAT CTT TCT ATT GCA TGG TGT AGG CTG GAG CTG CTT CG	*proVWX* (*proU*) deletion
DS078	CAA AAA CGC CTT ATC CGC CCG AAT AAA AAT TAC TTC TGC GCT GCC AGC GCC ATA TGA ATA TCC TCC TTA G	*proVWX* (*proU*) deletion
DS083	GAT CGA GCT CGT GCG TTG TTA TAT GAG CGT C	Construction of pMR20-ProQ
DS084	GAT CGG TAC CTC AGA ACA CCA GGT GTT CTG C	Construction of pMR20-ProQ
pBSIVTforward	CGT TGT AAA ACG ACG GCC AGT G	*proP* and *rpoS in vitro* transcription template
rpoSIVTreverse	GAT CTC ATT AGG TTG CGT ATG TTG AG	*rpoS in vitro* transcription template

### Preparation of cell lysates for ribosomal fractionation

Isolation of ribosomal species was performed as previously described [22] with the following exceptions. Cultures of LB broth were inoculated with 1/200^th^ volume of a stationary overnight culture. For strain JM6733 (Δ*proQ::KAN*) containing the indicated plasmids, Isopropyl β-D-1-thiogalactopyranoside (IPTG) was added to a final concentration of 15 µM after the first 30 min of incubation to induce the expression from the *lac* promoter. Cell pellets were resuspended in 700 µL of lysis buffer containing 10 mM Tris pH 7.5, 10 mM MgCl_2_, and 60 mM NH_4_Cl per 100 mL of starting culture, flash frozen in liquid nitrogen, and stored at -80°C. 300 µm glass beads were added to frozen cells as they thawed. Cells were lysed by repeated vortexing for 1 min followed by 1 min on ice for a total time of 10 min. The lysate was cleared by centrifugation at 21,000 x g for 10 min at 4°C. For subunit dissociation experiments, cells were lysed without addition of chloramphenicol, in buffer containing 10 mM Tris pH 7.5, 1 mM MgCl_2_, and 60 mM NH_4_Cl and clarified as described. The absorbances of clarified lysates were measured at 260 nm. 

### MNase digestion of ribosomes

Lysates were prepared as previously described. 13 OD_260_ units of lysate were transferred to a clean, 1.5 mL microcentrifuge tube. CaCl_2_ was added to a final concentration of 5 mM. BSA (NEB) was added to a final concentration of 0.1 mg/mL. 4 µL of MNase (NEB) were added and the final reaction volume was brought up to 400 µL with lysis buffer. Digests were carried out at room temperature for 30 min. Mock digests were set up as controls, containing all reagents except MNase.

### *In vitro* binding of ProQ and 70S ribosomes

 70S ribosomes (NEB) were incubated with an equimolar ratio of purified ProQ in polysome lysis buffer, with or without equimolar P2 mRNA. Reactions were loaded onto sucrose gradients and separated as described.

### Ribosomal fractionation

Ribosomal species were separated on sucrose gradients ranging from 10% to 47% sucrose in pollyalomar ultracentrifuge tubes (Beckman). Sucrose was dissolved in the following buffer: 100 mM NH4Cl, 10 mM Tris pH 7.5, 10 mM MgCl_2_. 550 µL of 47% sucrose was added into the bottom of the tube and placed at -80°C until frozen. Next, 42% sucrose was added and the tube was placed back at -80°C until frozen. This method was repeated until the gradient was complete, using the following concentrations of sucrose: 47%, 42%, 37%, 32%, 27%, 22%, 17%, 12%, 10%. Gradients were stored at -80°C. Gradients were allowed to thaw completely at room temperature. 13 OD_260_ units of lysate were applied to the top of each gradient. Gradients were balanced and submitted to ultracentrifugation in an SW50.1 (Beckman) rotor at 41K RPM for 1.5 h at 4°C. After ultracentrifugation, fractionation was performed as previously described [22]. Separation of dissociated subunits was done by ultracentrifugation on 20% sucrose cushions in an SW40TI (Beckman) rotor at 23 K RPM for 15 h at 4°C. Fractionation was performed as previously described.

### Mutant His-ProQ constructs

Mutant constructs were made using pCA24N-ProQ from the ASKA collection [23] as a template for inside-out PCR. A *Sac*I restriction endonuclease site was added to both primers (Table 2), allowing linear products from PCR amplification to be digested and self-ligated in frame to create pDS1-pDS5 (Table 1).

### ProQ purification and Antibody production

A culture of JM6733 (Δ*proQ::KAN*), transformed with pCA24N-ProQ, was grown to late-logarithmic growth phase (OD_600_^≈^ 0.8) at 37°C in LB media. Production of a 6 histidine epitope-tagged ProQ was induced by addition of IPTG to a final concentration of 1mM, and the culture was shifted to ambient temperature with shaking. Cells were harvested 18 h post induction. Cells were resuspended in 8 mL of lysis buffer (10% glycerol, 20 mM Tris pH 8.0, 500 mM NaCl, 10 mM Imidazole, and 1 mM β-Mercaptoethanol) and complete EDTA-free protease inhibitors (Roche) per 1 g of wet pellet weight and disrupted by 5 passes through a french press. Insoluble debris was cleared via centrifugation at 15,000 RPM for 30 min at 4°C in an SA-600 rotor (Sorvall). Nickel affinity chromatography was performed using Ni-NTA Superflow resin (Qiagen) as per manufacturer’s directions with the following exceptions. After batch binding of 6His-ProQ the column was washed with 5 column volumes (CVs) of lysis buffer. Additional 5 CV washes were done with lysis buffer containing 1 M NaCl, as well as lysis buffer containing 20 mM and 30 mM Imidazole. 6His-ProQ was eluted from the column using 5 CVs of lysis buffer containing 500 mM Imidazole. Protein purity was monitored via SDS-PAGE, and the eluent was dialyzed overnight at 4°C in a low-salt buffer (10% glycerol, 20 mM Tris pH 8.0, 100 mM NaCl, 1 mM β-Mercaptoethanol). Cation exchange chromatography was performed using SP Sepharose Fast Flow resin (GE Healthcare). Cation exchange resin was equilibrated with 5 CVs of low salt buffer. Dialyzed 6His-ProQ was incubated with equilibrated resin for 30 min at room temperature with occasional agitation. After batch binding, the column was washed with 5 CVs of low salt buffer. 6His-ProQ was eluted from the column using a gradient of NaCl (100 mM to 1 M). 6His-ProQ purity was monitored via SDS-PAGE, and purified 6His-ProQ was again dialyzed against a low-salt buffer. After dialysis, 6His-ProQ was concentrated using a centrifugal filter with a 3 kDa molecular weight cutoff (Amicon), as per manufacturer’s directions. Purified 6His-ProQ was quantified using an ND-1000 spectrophotometer (NanoDrop Technologies, Inc.) and 600 µg was sent for antibody production in a rabbit host (Cocalico Biologicals, Inc).

### Western-blot analysis of ribosomal fractionations

Denaturing SDS-PAGE loading buffer (50 mM Tris pH 6.8, 2% Sodium dodecyl sulfate (SDS), 10% glycerol, 1% β-Mercaptoethanol, 13 mM EDTA, 0.02% Bromophenol blue) was added to trichloroacetic acid (TCA) precipitated ribosomal fractions. Briefly, TCA was added to polysome gradient fractions to a final concentration of 15% and incubated at 4°C for 30 min. The precipitant was pelleted in a microcentrifuge at maximum speed for 10 min. The supernatant was removed and the pellet was washed twice with ice cold 100% acetone. Proteins were separated via SDS-PAGE and transferred to nitrocellulose (GE Water & Process Technologies). Protein detection was carried out using antibodies against ProQ (1:5000 in 5% dry milk in PBS + 0.1% Tween20) and ribosomal proteins L3 (1:5000 in same) and S2 (1:5000 in same). Antibodies to L3 and S2 were a generous gift from Catherine Squires.

### Copy number calculation

*proQ* was cloned into vector pMCSG7 as described [24] using DS046 and DS047 (Table 2) to make pDS6 (Table 1), which adds an N-terminal 6-Histidine epitope tag, followed by a TEV cleavage site. ProQ was purified as previously described with the following exceptions. After the first nickel affinity column, elution fractions were pooled and dialyzed overnight in the presence of 6His-TEV protease. A second nickel affinity column was used to remove TEV protease and the flow through was collected. Fractions were analyzed via SDS-PAGE and pooled for cation-exchange chromatography. Purified ProQ was quantified using a Bradford assay (Biorad). Known amounts of ProQ, as well as whole cell lysate from a known number of wild type BW25113 *E. coli* cells were loaded onto SDS-PAGE and transferred to nitrocellulose for western-blot analysis using anti-ProQ antibodies. Band intensities were quantified using ImageJ and cellular concentration was calculated.

### In vitro transcription

The *proP* genomic region was cloned into pBluescriptKS+ in two forms, starting at either -182 (*proP* P1, DS037, DS039) or -95 (*proP* P2, DS038, DS039). The *rpoS* genomic region was cloned into pBluescriptKS+ (rpoS-T7-forw_SacII, rpoS-T7-rev-PstI). PCR was performed to amplify the plasmid DNA for use as an *in vitro* transcription template. The forward primer maintained the integrity of the T7 polymerase dependent promoter (pBSIVTforward), and the reverse primer added a stop codon after glycine 245 for *proP* P1and P2 (DS076) and after tyrosine 147 for *rpoS* (rposIVTreverse), yielding mRNAs with sizes of 927 bp, 840 bp, and 1041 bp respectively. *In vitro* transcription was carried out as described previously [25], and the mRNAs were checked for quality using urea-acrylamide gel electrophoresis and TAE-agarose electrophoresis. The mRNA concentrations were determined by electrophoresis on a TAE-agarose gel and quantitative comparison to the RiboRuler High Range RNA Ladder (Thermo Scientific).

### Tryptophan fluorescence

Purified ProQ was dialyzed into 10% glycerol, 20 mM HEPES pH 7.5, and 100 mM NaCl. The concentration was determined using Bradford assay. For binding experiments, ProQ was diluted to a final concentration of 20 nM in the same buffer plus 0.005% BRIJ35. Temperature was held constant at 20°C and tryptophan was excited at 295 nm and fluorescence was monitored at 355 nm using a QuantaMaster4 (Photon Technology International). The excitation wavelength was chosen to avoid any possible inner-filter effect stemming from the use of nucleic acids as ligands [26]. Increasing amounts of *in vitro* transcribed mRNAs were added to the reaction and the change in fluorescence was observed.

### Biofilm assays

Plasmid pMR20-ProQ was constructed by cloning the amplicon from primers DS083 and DS084 into pMR20 [27] cut with *Kpn*I and *Sac*I (Table 2). Biofilm assays were performed with modifications of previous protocols [28,29] as follows. Strains were grown overnight in LB + 12.5 µg/mL tetracycline. 30 µL of the saturated overnight was inoculated into 1 mL of LB without salt (10 g tryptone, 5 g yeast extract) plus 12.5 µg/mL tetracycline, in a previously unused borosilicate test tube. Tubes were incubated at 25°C without shaking for 6 d. After 6 d, the media and loose cells were removed and the OD_600_ was measured. Tubes were gently rinsed by submerging in deionized water 5 times and allowed to air dry. Biofilms were stained using 1% crystal violet for 20 min. Tubes were again rinsed by submerging in deionized water to reduce background staining. Biofilms were resuspended completely in 0.5% SDS and the OD_600_ was measured. Relative biofilm formation was calculated as described previously [28].

## Results

### ProQ associates with translating ribosomes

**Figure 1:**
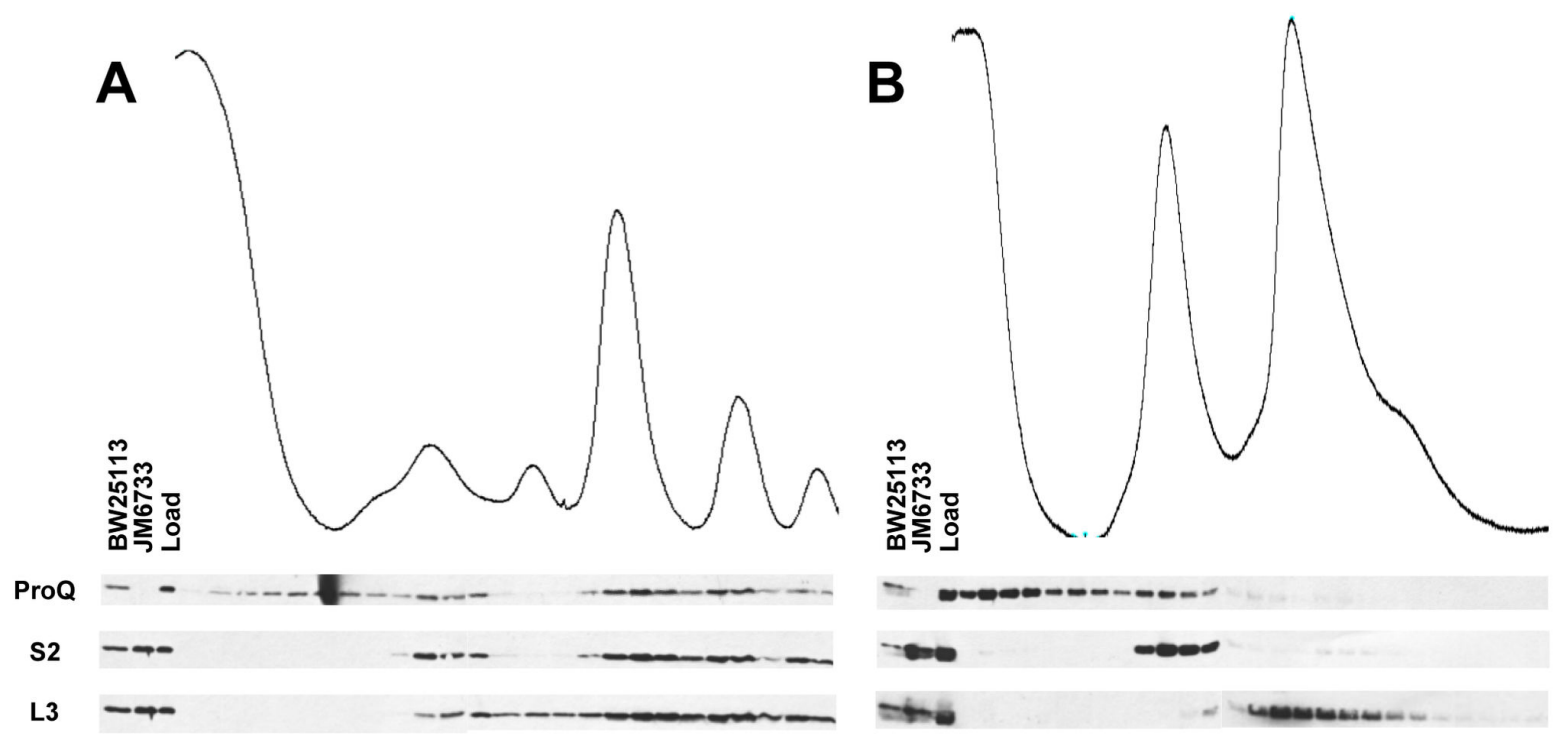
Ribosome association of ProQ under native and dissociating conditions. Polysome profiles (254 nm) from wild type cell extracts in (A) associating conditions (10 mM Mg^2+^) and (B) dissociating conditions (1 mM Mg^2+^) are shown. Western-blot analysis of TCA-precipitated fractions using antibodies to ProQ, small ribosomal subunit protein 2 (S2), and large ribosomal subunit protein 3 (L3) are shown and aligned to the UV-absorbance trace. Whole cell extracts from wild type (BW25113) and Δ*proQ* (JM6733) are included, as is the soluble lysate (Load). UV-absorbance peaks correspond to (L to R): Free RNA/protein, 30S, 50S, 70S, and polysomes.

In order to verify the localization of ProQ on ribosomes, the soluble lysates from wild type *E. coli* (strain BW25113) were separated on sucrose density gradients (Figure 1A). Western-blot analysis of the resulting fractions revealed that ProQ predominantly associates with 70S and translating ribosomes, and to a lesser extent, at the top of the gradient and in fractions corresponding to the 30S ribosomal subunit. Antibodies against small and large ribosomal subunit proteins validated the assignment of the peaks in the UV-absorbance trace. Deletion of some ribosome-associated factors can lead to ribosomal defects and perturbation of the polysome profile [22]. Profiles from a Δ*proQ* strain were indistinguishable from wild type (data not shown), and therefore, ProQ is not likely a ribosome assembly factor, nor is it required for the translation of the majority of mRNAs in the cell.

Since the 30S and 50S ribosomal subunits work in tandem, but with separate roles during translation, identifying the subunit with which ProQ associates provides a clue as to the function of ProQ during translation. When isolated at lower concentrations of Mg^2+^, 70S ribosomes dissociate into 30S and 50S subunits [30]. At 1 mM Mg^2+^, the 70S and polysome species were predominantly dissociated, as revealed by the UV-absorbance trace and localization of S2 and L3 (Figure 1B). A large proportion of ProQ was found at the top of the gradient, but ProQ that did migrate into the gradient peaked with the 30S subunit but not with the 50S subunit. These data are consistent with the localization under non-dissociating conditions (Figure 1A). Thus, ProQ appears to preferentially associate with the 30S subunit.

### The N-terminus and linker regions are necessary for ribosome association

**Figure 2:**
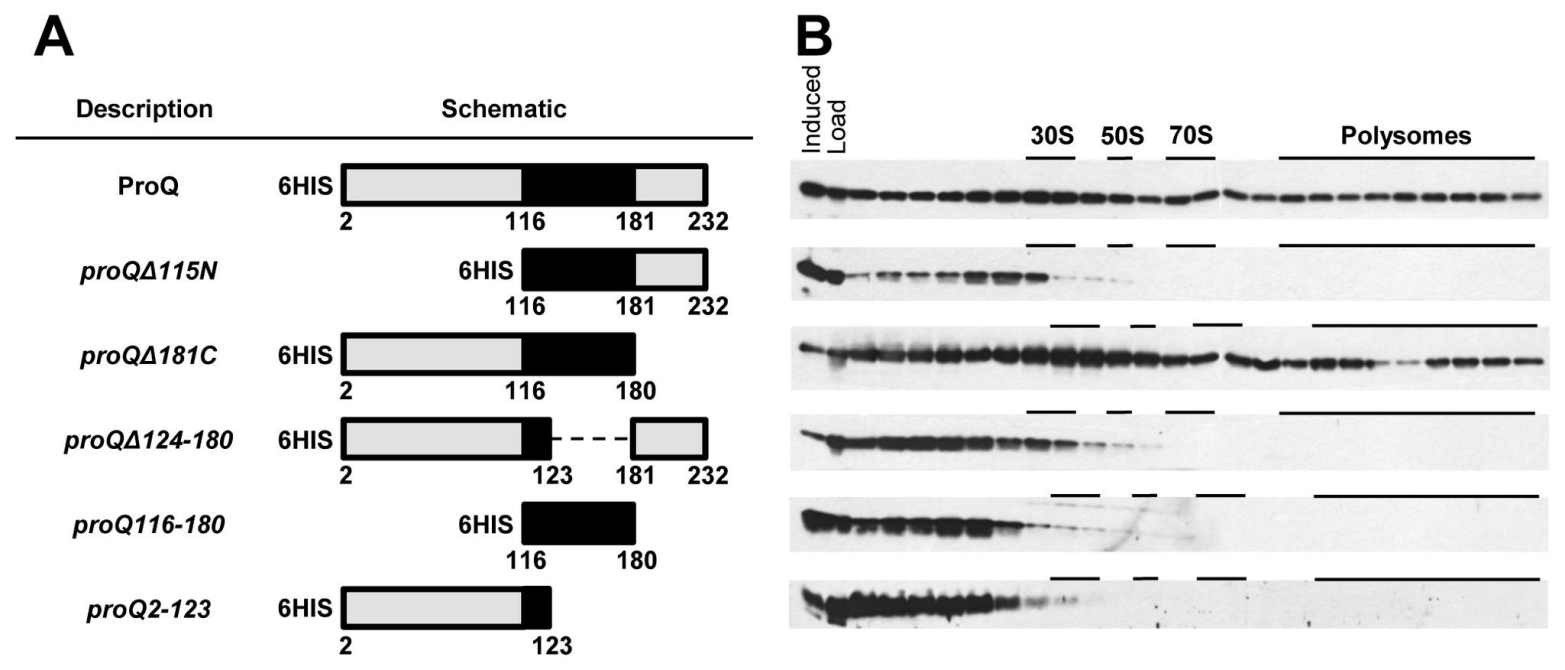
Ribosome association of plasmid encoded *proQ* mutant constructs in polysome profiles. (A) Schematic representation of *proQ* mutant constructs expressed from the IPTG inducible plasmid pCA24N. The predicted RNA binding region is colored in black, and amino-acid boundaries of each construct are labeled. (B) Mutant constructs were expressed as the lone copy of *proQ*. Western-blot analysis of TCA-precipitated fractions from the separation of ribosomal moieties on sucrose density gradients was performed. Whole cell extracts of cells after induction were included (Induced) along with soluble lysate (Load). Position of the 30S, 50S, 70S, and Polysome species are indicated based on western-blot localization of S2 (data not shown).

ProQ is predicted to contain two structural domains, tethered by a positively charged linker region. The N- and C-termini have been modeled on the RNA binding proteins FinO and Hfq respectively [12,15]. Using available bioinformatic tools to predict secondary structure, disordered regions, and nucleic acid binding propensity [31–33], we assigned the boundaries of the linker region as amino acids E116-V180. Assignment of the lower bound was the most difficult. The nucleic acid binding predictor, BindN, indicated the presence of an unstructured positively charged domain starting at E116. The secondary structure predictor, PSIPRED, indicated the presence of an alpha helix until A124. Domain constructs were made using the 6HIS-ProQ plasmid from the ASKA collection [23] as a template. A schematic of each construct is provided (Figure 2A). To determine the regions of ProQ that are necessary for its association with the ribosome, each ProQ construct was expressed in a Δ*proQ* background, such that it was the only copy of ProQ in the cell. We could not detect a ProQ variant expressing only the C-terminal domain (*proQ181-232*). The distribution of ectopically expressed 6HIS-ProQ (Figure 2B) was similar to that of ProQ from wild type cells (Figure 1A); although, in addition to an association with 70S, polysome, and 30S fractions, there was a general increase in ProQ throughout the gradient. This broader distribution may reflect a higher than normal cellular concentration caused by exogenous expression or a decrease in affinity for translating ribosomes due to the N-terminal 6HIS-epitope tag. The high rate of speed and high G-forces produced during polysome fractionation can physically disrupt binding interactions, so addition of the epitope tag may cause dissociation of 6HIS-ProQ from ribosomes as it progresses through the gradient. Deletion of the C-terminal Hfq domain (*proQΔ181C*) did not affect association with the ribosomes (Figure 2B), and therefore, this part of the protein is not required for ribosome localization. In contrast, both the N-terminus and linker regions are required for ribosome association, as the majority of *proQΔ115N* and *proQΔ124-180* were found at the top of the gradient. Consistently, removal of the C-terminus and linker domains together (*proQ2-123*) caused ProQ to be found at the top of the gradient. Moreover, the linker domain (*proQ116-180*) was not sufficient for ribosome association. These data suggest that both the N-terminal FinO domain and the linker regions are required for ribosome association of ProQ.

### ProQ interacts with the mRNA being translated

**Figure 3:**
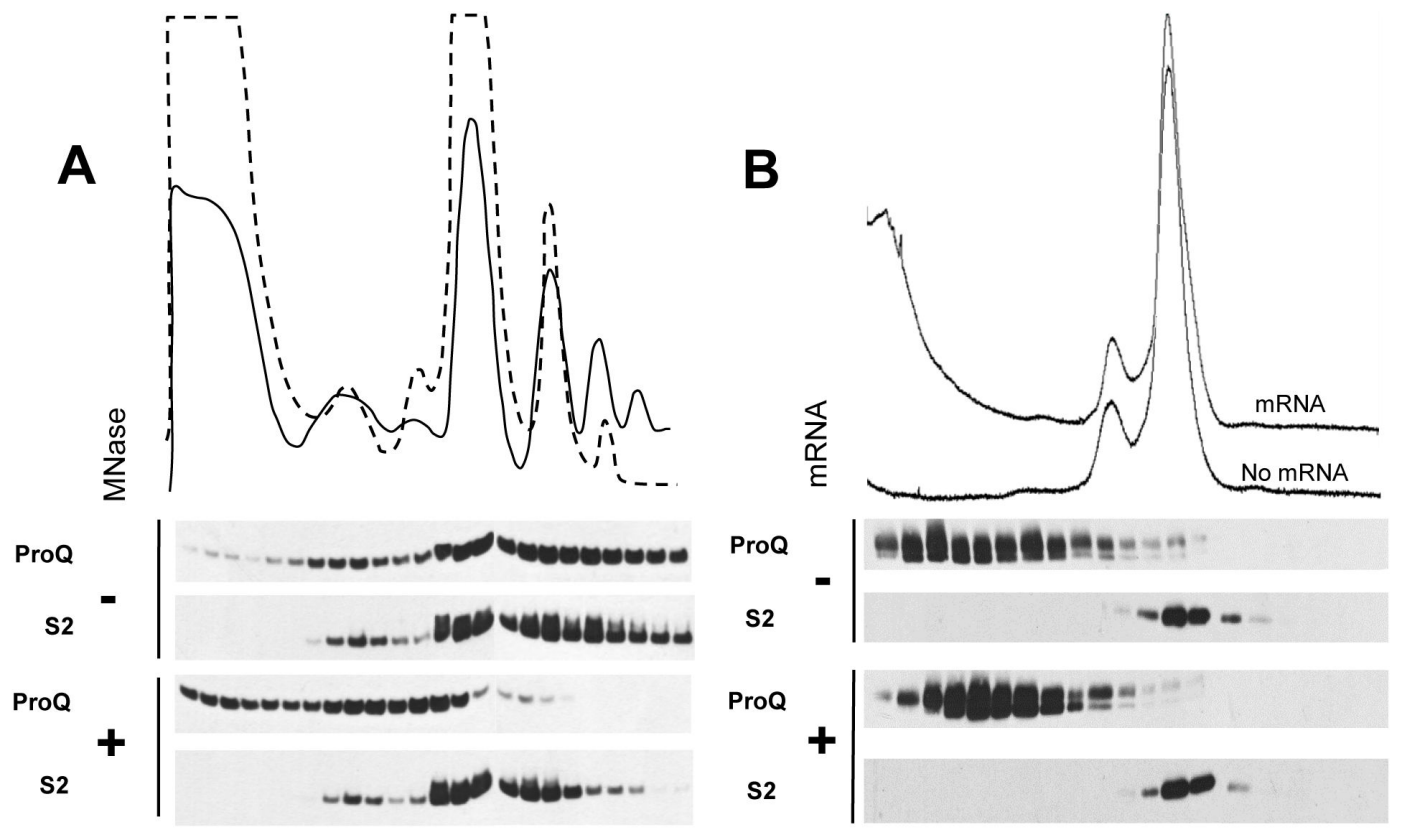
Ribosome association of ProQ after mRNA disruption. (A) Cell lysates were left untreated (solid line) or treated with limited MNase (dashed line) and ribosomal species separated on sucrose gradients. The positions of ProQ and ribosomal protein S2 in the resulting TCA-precipitated fractions is determined by western-blot analysis. (B) mRNA free 70S ribosomes were mixed with equimolar amounts of purified ProQ without (foreground) or with (background) the addition of equimolar P2 mRNA before application to sucrose gradients. The position of ProQ and ribosomal protein S2 in the resulting TCA-precipitated fraction is determined by western-blot analysis.

The comigration of ProQ into sucrose gradients with translating ribosomes could occur because of a physical interaction with the ribosome directly or because of an interaction with the mRNA bound by a 30S particle and/or 70S particle. To determine the nature of the ProQ association with ribosomes, we partially hydrolyzed the mRNA in cell lysates with micrococcal nuclease (MNase) under conditions that did not perturb the integrity of the highly structured, and relatively RNase insensitive, ribosome. As expected, the small ribosomal protein S2 was found associated with the remaining 70S and 2-mers, and no appreciable amount of S2 was found at the top of the gradient. This supports the claim that the integrity of these ribosomes was maintained. In mock treatments, mRNAs with up to 4 ribosomes are seen in sucrose gradients, and ProQ associates with all translating ribosomes (both the 70S and polysome forms) (Figure 3A). After treatment with MNase, the level of total polysomes was decreased concomitant with a large increase in free 70S ribosomes (Figure 3A) and consistent with cleavage of intra-ribosome mRNA. Strikingly, in contrast to a robust association of ProQ with 2-mer and 3-mer polysomes in untreated samples, ProQ was almost absent from these particles in the MNase treated samples. Additionally, a significant amount of ProQ was found at the top of the gradient, consistent with dissociation of ProQ from the ribosomes after disruption of mRNA. Thus, ribosome association of ProQ appears to be partially dependent on the mRNA being translated. In order to support this finding, we also performed *in vitro* binding experiments with mRNA-free 70S ribosomes. 70S ribosomes were incubated with equimolar purified ProQ, with or without *proP* P2 mRNA. After incubation, the reactions were applied to sucrose gradients and separated as before (Figure 3B). Though some ProQ is found in the gradient under the 70S peak, indicating a weak interaction with 70S ribosomes, the bulk of the ProQ is located in the fractions not corresponding to the 70S ribosomes. It is also worth noting that when *proP* mRNA was added, we consistently observed the concentration of ProQ in the gradient peaking around fraction 5. The most likely explanation for this result is that ProQ is binding to the *proP* mRNA and migrating to this region of the gradient.

### ProQ does not bind selectively to *proP* mRNA

**Figure 4:**
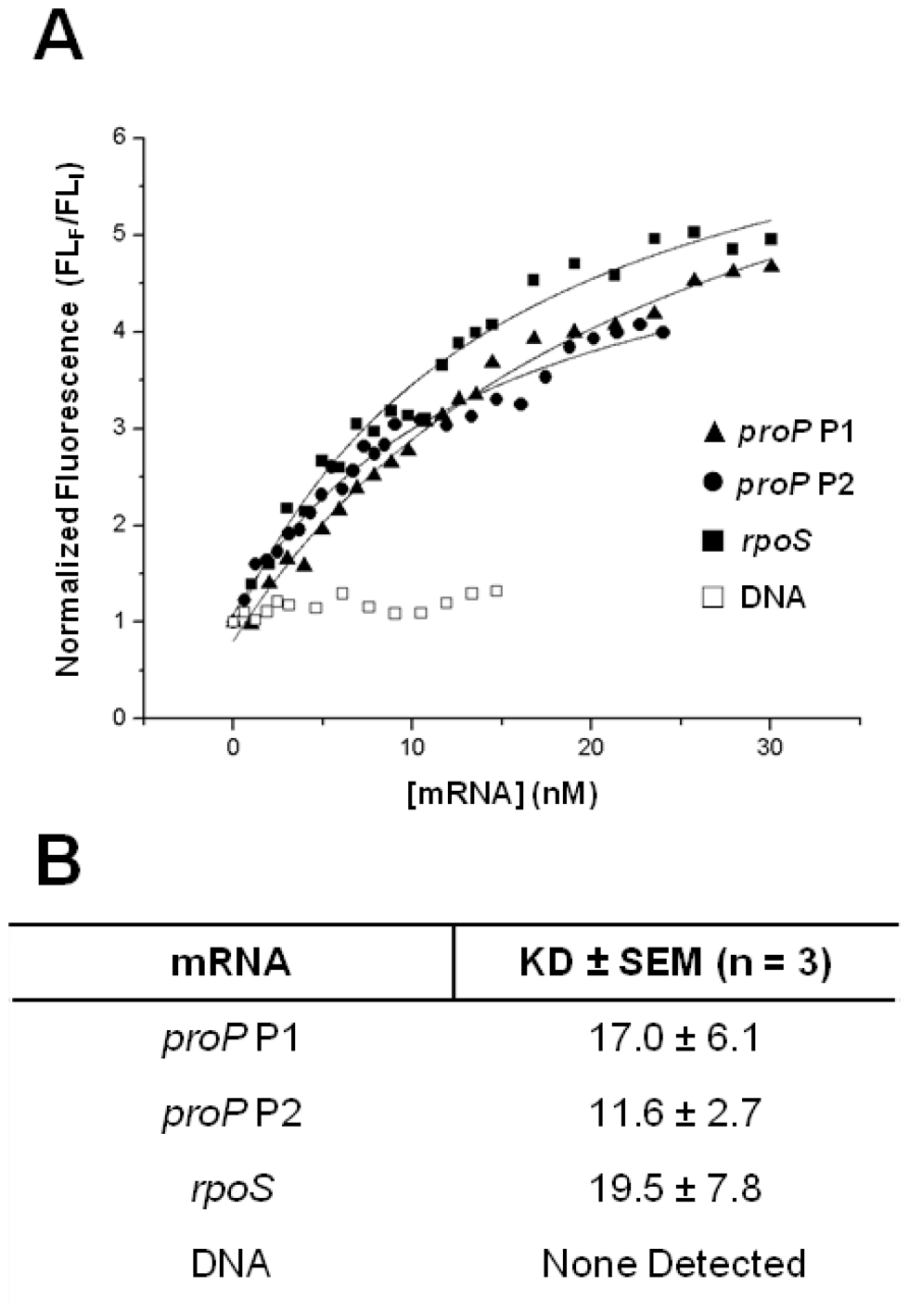
mRNA binding kinetics monitored by native tryptophan fluorescence. (A) 20 nM ProQ was titrated with increasing amounts of *in* *vitro* transcribed mRNAs (“●” *proP* P1, “▲” *proP* P2, “■” *rpoS*). Tryptophan was excited at 295 nm and the fluorescence was monitored at 355 nm. The y-axis is provided in terms of normalized fluorescence (FL_F_/FL_I_), determined by dividing the fluorescence at each mRNA concentration (FL_F_) by the fluorescence for 20nM protein only (FL_I_). DNA yielded no measurable fluorescence shift and is included as a negative control (“□” DNA) (B) Summary of binding affinities of ProQ for *in* *vitro* transcribed mRNAs.

It has been demonstrated that ProQ can bind to a model RNA substrate *in vitro* [15]. However, the model double-stranded substrate used previously was relatively small (39 bp in duplex form) compared to the size of an mRNA. To further understand the RNA binding properties of ProQ, binding experiments were performed using purified ProQ and *in vitro* transcribed mRNA substrates (Figure 4A). One possibility is that ProQ acts as a *proP* mRNA-specific translation factor. If true, we would predict that ProQ should interact preferentially with *proP* mRNA *in vitro*. We tested the ability of ProQ to bind to mRNA made from the *proP* P1 and P2 promoters, as well as an unrelated mRNA, *rpoS* (see methods). Under the conditions tested, ProQ showed no preference for *proP* mRNA transcribed from promoter P1 or P2, and the binding affinity for *proP* P1 and P2 were similar (17.0 ± 6.1 nM and 11.6 ± 2.7 nM, respectively; Figure 4). Interestingly, ProQ also bound tightly to *rpoS* mRNA (19.5 ± 7.8 nM; Figure 4). These slight differences among the *K*_*D*_ values are not sufficient to confer selectivity *in vivo*. Comparisons of our values to those previously reported for the model substrate are in agreement [15], though we report consistently lower *K*_*D*_ values. ProQ binds non-selectively to all mRNAs tested, but it does not bind to DNA, and we include the results as a negative control. To compliment the determination of the *K*_*D*_ values for specific mRNAs we sought to determine the cellular abundance of ProQ. Using quantitative western-blot analysis, we estimate the cellular copy number of ProQ to be 1.90 x 10^3^ ± 324 (95% confidence interval, data not shown). Assuming a cellular volume of 1.3 µm^3^ [34], the cellular concentration of ProQ is 2.43 ± 0.414 µM. This number is comparable to another estimate for the ProQ copy number obtained by high-throughput proteomic analysis of *E. coli* cytosolic proteins [35], and at approximately 2,000 copies/cell, ProQ is in the top 25% in terms of protein abundance.

### Ribosome association of ProQ is not dependent on proP or proU

**Figure 5:**
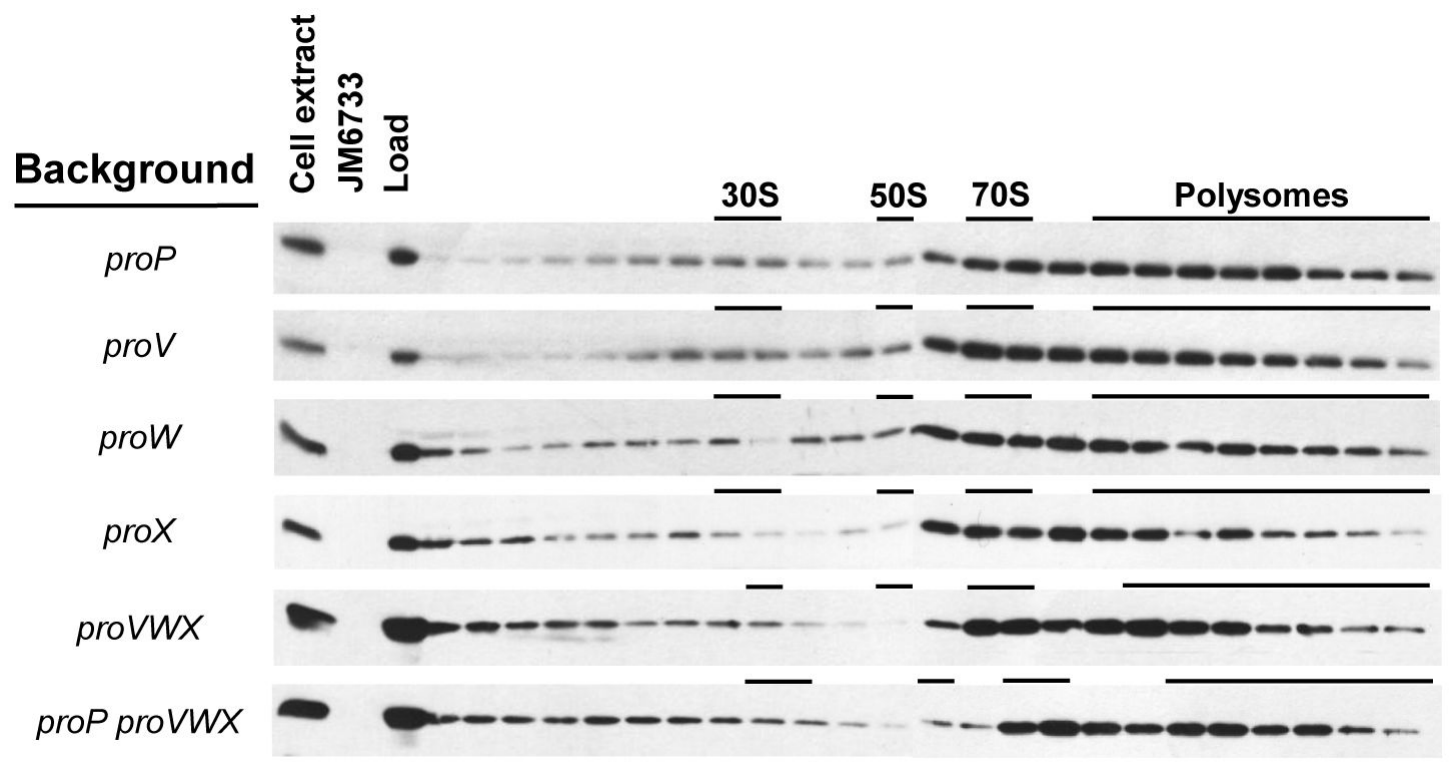
Ribosome association of ProQ in various strain backgrounds. Cell lysates from various mutant backgrounds, as indicated, were separated by sucrose density ultracentrifugation and the localization of the ribosomal species (30S, 50S, 70s, polysomes) indicated. The localization of ProQ in the TCA-precipitated fractions is shown by immunoblot. Whole cell extracts from wild type, Δ*proQ* (JM6733), and the soluble lysate (Load) are included as indicated.

We have shown that ProQ is found on translating ribosomes and this association appears to be mediated through mRNA. Because the only well characterized phenotype for a *proQ* mutant is a decrease in proline uptake, we posit that ProQ is involved in the translation of a subset of mRNAs in the cell, including one or more of the proline transporter mRNAs. We therefore asked whether the ribosome association of ProQ was dependent on these mRNA transcripts. We first examined the localization of ProQ in a Δ*proP* mutant, as *proP* mRNA is the predicted target of ProQ. We found, however, that the ribosome association of ProQ was similar in a Δ*proP* strain to that seen in the wild type strain (Figures 1 and 5). A genetic interaction has been reported between *proQ* and the *proW* and *proX* loci within the *proU* operon [36] and therefore, these genes may be regulated by ProQ. For this reason, we examined ProQ localization in strain backgrounds deleted for each member of the *proU* operon, as well as a deletion of the entire *proU* operon (*proVWX*), and deletion of both *proVWX* and *proP* together. In all backgrounds tested, ProQ is repeatedly found associated with 70S particles and polysomes (Figure 5). Thus, ProQ ribosome association is not absolutely dependent on any of these genes, and the ribosome association of ProQ may be due to one or more additional cellular mRNAs. 

### ProQ is necessary for biofilm formation, independent of ProP

**Figure 6:**
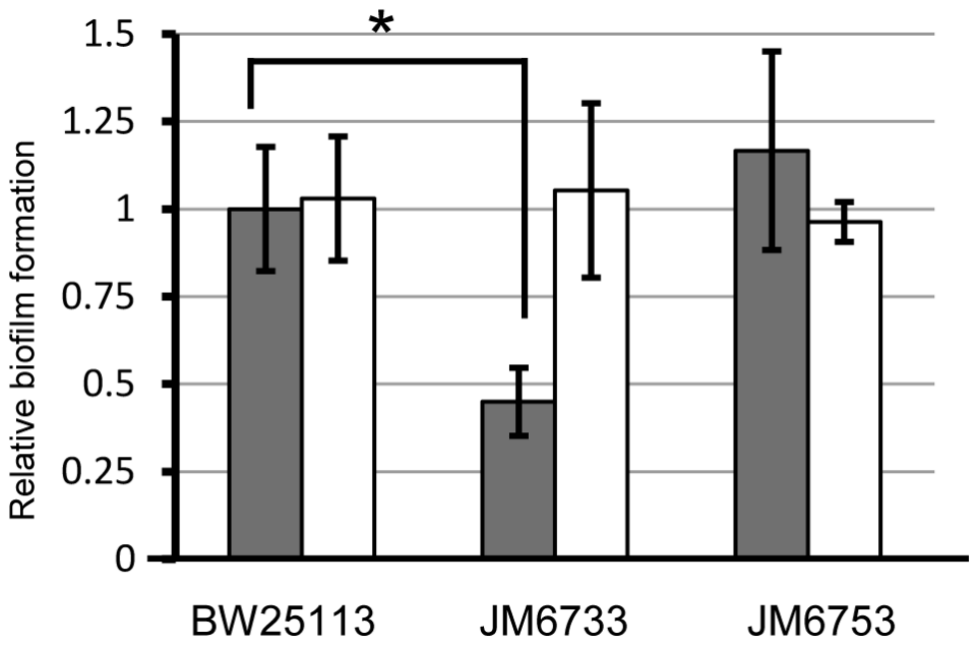
Biofilm formation defect in a Δ*proQ* strain. Wild type (BW25113), Δ*proQ* (JM6733), and Δ*proP* (JM6753) strains were examined for formation of biofilms after 6 days. Strains were transformed with either empty vector (pMR20; shaded) or vector containing the *proQ* open reading frame, plus 500 bp of genomic sequence upstream of the translation start site (pMR20-ProQ; unshaded). Error bars represent 95% confidence intervals. A statistical difference is indicated (*) between wild type and Δ*proQ* (JM6733) with a p-value < 0.001.

Having shown that ProQ localization to the ribosome is independent of all known potential interactions involved in proline transport, we sought to expand the ProQ target list by looking for new Δ*proQ* phenotypes. A high throughput study implicated ProQ in biofilm formation; though, it only reported it as a hit and did not verify the result through plasmid-based complementation [28]. Under the conditions tested, a *proQ* mutant strain was found to be about 50% deficient in biofilm formation compared to wild type cells (Figure 6). This phenotype was complemented by transforming Δ*proQ* cells with a low-copy plasmid containing the *proQ* gene, plus 500 bp of sequence upstream of the translation start site. To test if this phenotype was independent of the role of ProQ in ProP regulation, we examined biofilm formation in a *proP* mutant strain. This strain was able to form biofilms as well as wild type. As a result, we conclude that the decrease in biofilm formation is unique to a *proQ* mutant and is independent of the ProQ-ProP interaction, though the reason for this deficiency remains unclear. 

## Discussion

It has been proposed that ProQ is a translational regulator of *proP* mRNA [15]. This hypothesis has evolved and emerged out of a sort of “process of elimination”, whereby it has been shown that i) *proP* transcript levels are unchanged in a *proQ* mutant [11] ii) no physical interaction can be detected between ProQ and ProP [36], and recently iii) ProP expression levels are affected by deletion of *proQ* [15]. In this study, we present the first *direct* evidence of a ProQ-translation link by demonstrating the association of ProQ with 70S particles, translating ribosomes, and 30S particles (Figure 1), but we have not detected a specific interaction between ProQ and *proP* mRNA at the ribosome. Some proteins that associate with the ribosome are involved in ribosome maturation and assembly [22,37]. Deletion of these factors can result in defects in polysome profiles and decreased growth rates [for reviews see 38]. These phenotypes are not observed in a *proQ* mutant (data not shown), and because of the relative dearth of phenotypes associated with a *proQ* deletion, it is unlikely that ProQ is an assembly factor. Thus far, ProQ has only been implicated in proline uptake [11] and stimulation of biofilm formation [[28] and this study]. For these reasons, we propose that ProQ has a very narrowly defined role in the translation of only a subset of mRNAs. This hypothesis is bolstered by the fact that the N-terminus of ProQ has been modeled on FinO, a highly specified translational regulator, which acts to specifically represses the translation of *traJ* mRNA by facilitating the interaction between *traJ* and the anti-sense RNA *finP* [39,40]. As for the identities of the specific mRNA targets of ProQ, *proP* remains a favored target, but it is unknown if this effect is direct or indirect. Additionally, one or more additional targets are predicted to exist, based on the ProP-independent biofilm defect in a *proQ* mutant.

It had previously been shown that ProQ could bind to a model RNA, and that ProQ exhibits RNA chaperone-like activity [15]. Here we sought to explore the interaction between ProQ and RNA in three distinct ways. First, we examined the consequences of nuclease treatment on the association of ProQ with the ribosome (Figure 3A). Limited treatment of lysates with MNase caused a decrease in the number of polysomes observed after centrifugation. Specifically, the number of 3-mers and higher molecular weight species were significantly decreased. The loss of 3-mers is consistent with the increase in the number of 70S ribosomes observed. Though 3-mers were almost completely missing, a significant amount of ribosomes were still found to exist as 2-mers. MNase treatment had a large effect on the localization of ProQ, however, causing it to almost completely delocalize from 2-mers and 70S ribosomes. To further explore the requirement of mRNA for ProQ-ribosome association, we examined the ability of ProQ to bind to mRNA-free ribosomes *in vitro*. Not surprisingly, ProQ did not robustly associate with these ribosomes, and instead, seemed to prefer to bind to mRNA when present in the reactions (Figure 3B). For these reasons, we conclude that the mRNA being translated is very important for the comigration of ProQ with 70S and polysome species. Next, we used a quantitative *in vitro* binding assay to determine the affinity of ProQ for its predicted mRNA target, *proP* (Figure 5). The hypothesis that ProQ enhances *proP* translation, to the exclusion of other mRNAs, leads to the prediction that ProQ should selectively recognize *proP*. Under the conditions tested, ProQ binds tightly to *proP* mRNAs, but this binding is not selective, as ProQ binds with similar affinity to *rpoS* mRNA (Figure 4). Though we cannot rule out the possibility that *rpoS* is also a target of ProQ regulation, we believe this is unlikely since RpoS is a known regulator of *proP* transcription [6,7], *proP* transcript levels are unchanged in a ProQ mutant [11], and a *proQ* deletion strain does not have the same lack of thermotolerance seen in an *rpoS* deletion strain [15]. If *proP* is a direct target of ProQ, some other unknown factor must confer specificity *in vivo*. 

Little is known about ProQ, mechanistically. Previous studies have shown that exogenous expression of the N-terminal, FinO-like domain (residues 1-130) can partially complement the proline uptake deficiency of a *proQ* chromosomal deletion, and the N-terminus (1-130) is necessary and sufficient for binding to RNA [13,15]. Our study of the domains of ProQ involved in ribosome association is not entirely consistent with these previous results. For example, even though the first 130 residues could bind to a model RNA, we do not observe ribosome association of a similar construct to any appreciable degree (Figure 2B). In this study, we expand the linker by seven residues to include amino acids 124-180, thus shortening the N-terminal domain from 1-130 to 1-123 (Figure 2A). This difference could account for the lack of ribosome association if residues 124-130 are necessary for RNA binding. Another inconsistency is observed with the C-terminus. In this study we find that a construct lacking residues 181-232 is associated with the ribosome in a manner comparable to plasmid-expressed wild type ProQ (Figure 2B). However, in previous studies, this construct did not suppress the proline uptake deficiency as well as a construct containing the C-terminal domain [13]. It must be noted that our present study sought only to identify the domains of ProQ which are important for ribosome association, independent of *proQ-proP* genetic interactions. Based on our results, we conclude that only the C-terminus is dispensable for ribosome association. Though we would predict that ribosome association is necessary for suppression of the proline uptake phenotype, we do not observe a direct dependence. This further complicates the mechanism by which ProQ enhances proline uptake.

A deeper exploration of the mechanism of ProQ action will be predicated on the discovery of *direct* mRNA targets. To date, finding these targets has been challenging. To begin the process, here we verified that ProQ is involved in promoting biofilm formation [28]. More importantly, we show that this phenotype is independent of ProP, yielding the first ProP-independent ProQ phenotype (Figure 6). Further independence of ProQ from ProP is demonstrated by the fact that ProQ is still associated with ribosomes in the absence of *proP* and *proU* mRNAs (Figure 5).

Though further studies are needed to definitively determine the function of the ribosomal association of ProQ, it is not unreasonable to propose a role for ProQ in translation initiation. Under non-dissociating conditions, we observe the highest concentration of ProQ in fractions corresponding to 70S and polysome species, but there is a modest, yet consistent, increase in signal under the peak corresponding to 30S particles, the first particle to bind during initiation (Figure 1A). This 30S localization is confirmed under dissociating conditions (Figure 1B). We present data showing that ProQ can bind tightly to mRNA *in vitro* (Figure 4), and mRNA integrity is necessary for robust ribosome association (Figure 3). Thus, ProQ is ideally positioned to function in translation initiation of its mRNA targets, perhaps by facilitating the interaction between 30S particles and these as-yet unidentified mRNAs.

## References

[1] Wood JM (2006) Osmosensing by bacteria. Sci STKE: 2006: pe43 10.1126/stke.3572006pe43 17047223

[2] MacMillan SV, Alexander DA, Culham DE, Kunte HJ, Marshall EV et al. (1999) The ion coupling and organic substrate specificities of osmoregulatory transporter ProP in Escherichia coli. Biochim Biophys Acta 1420: 30-44. doi:10.1016/S0005-2736(99)00085-1. PubMed: 10446288. 10446288

[3] Milner JL, Grothe S, Wood JM (1988) Proline porter II is activated by a hyperosmotic shift in both whole cells and membrane vesicles of Escherichia coli K12. J Biol Chem 263: 14900-14905. PubMed: 3049595. 3049595

[4] Wood JM (1999) Osmosensing by bacteria: signals and membrane-based sensors. Microbiol Mol Biol Rev 63: 230-262. PubMed: 10066837. 10066837 10.1128/mmbr.63.1.230-262.1999 PMC98963

[5] Mellies J, Wise A, Villarejo M (1995) Two different Escherichia coli proP promoters respond to osmotic and growth phase signals. J Bacteriol 177: 144-151. PubMed: 8002611. 8002611 10.1128/jb.177.1.144-151.1995 PMC176566

[6] Xu J, Johnson RC (1995) Fis activates the RpoS-dependent stationary-phase expression of proP in Escherichia coli. J Bacteriol 177: 5222-5231. PubMed: 7545153. 7545153 10.1128/jb.177.18.5222-5231.1995 PMC177312

[7] Xu J, Johnson RC (1997) Activation of RpoS-dependent proP P2 transcription by the Fis protein in vitro. J Mol Biol 270: 346-359. doi:10.1006/jmbi.1997.1133. PubMed: 9237902. 9237902

[8] McLeod SM, Aiyar SE, Gourse RL, Johnson RC (2002) The C-terminal domains of the RNA polymerase alpha subunits: contact site with Fis and localization during co-activation with CRP at the Escherichia coli proP P2 promoter. J Mol Biol 316: 517-529. doi:10.1006/jmbi.2001.5391. PubMed: 11866515. 11866515

[9] Xu J, Johnson RC (1997) Cyclic AMP receptor protein functions as a repressor of the osmotically inducible promoter proP P1 in Escherichia coli. J Bacteriol 179: 2410-2417. PubMed: 9079929. 9079929 10.1128/jb.179.7.2410-2417.1997 PMC178980

[10] Landis L, Xu J, Johnson RC (1999) The cAMP receptor protein CRP can function as an osmoregulator of transcription in *Escherichia* *coli* . Genes Dev, 13: 3081-3091. PubMed: 10601034. 10601034 10.1101/gad.13.23.3081 PMC317180

[11] Milner JL, Wood JM (1989) Insertion proQ220::Tn5 alters regulation of proline porter II, a transporter of proline and glycine betaine in Escherichia coli. J Bacteriol 171: 947-951. PubMed: 2536686. 2536686 10.1128/jb.171.2.947-951.1989 PMC209686

[12] Smith MN, Crane RA, Keates RA, Wood JM (2004) Overexpression, purification, and characterization of ProQ, a posttranslational regulator for osmoregulatory transporter ProP of Escherichia coli. Biochemistry 43: 12979-12989. doi:10.1021/bi048561g. PubMed: 15476391. 15476391

[13] Smith MN, Kwok SC, Hodges RS, Wood JM (2007) Structural and functional analysis of ProQ: an osmoregulatory protein of Escherichia coli. Biochemistry 46: 3084-3095. doi:10.1021/bi6023786. PubMed: 17319698. 17319698

[14] Ghetu AF, Gubbins MJ, Frost LS, Glover JN (2000) Crystal structure of the bacterial conjugation repressor finO. Nat Struct Biol 7: 565-569. doi:10.1038/76790. PubMed: 10876242. 10876242

[15] Chaulk SG, Smith Frieday MN, Arthur DC, Culham DE, Edwards RA et al. (2011) ProQ is an RNA chaperone that controls ProP levels in Escherichia coli. Biochemistry 50: 3095-3106. doi:10.1021/bi101683a. PubMed: 21381725. 21381725

[16] Kunte HJ, Crane RA, Culham DE, Richmond D, Wood JM (1999) Protein ProQ influences osmotic activation of compatible solute transporter ProP in Escherichia coli K-12. J Bacteriol 181: 1537-1543. PubMed: 10049386. 10049386 10.1128/jb.181.5.1537-1543.1999 PMC93544

[17] Jiang M, Sullivan SM, Walker AK, Strahler JR, Andrews PC et al. (2007) Identification of novel Escherichia coli ribosome-associated proteins using isobaric tags and multidimensional protein identification techniques. J Bacteriol 189: 3434-3444. doi:10.1128/JB.00090-07. PubMed: 17337586. 17337586 PMC1855874

[18] Schrenk WJ, Miller JH (1974) Specialized transducing phages carrying fusions of the trp and lac regions of the E. coli chromosome. Mol Gen Genet 131: 9-19. doi:10.1007/BF00269382. PubMed: 4605133. 4605133

[19] Baba T, Ara T, Hasegawa M, Takai Y, Okumura Y et al. (2006) Construction of Escherichia coli K-12 in-frame, single-gene knockout mutants: the Keio collection. Mol Syst Biol 2: 0008. PubMed: 16738554 10.1038/msb4100050 PMC1681482 16738554

[20] Datsenko KA, Wanner BL (2000) One-step inactivation of chromosomal genes in Escherichia coli K-12 using PCR products. Proc Natl Acad Sci U S A 97: 6640-6645. doi:10.1073/pnas.120163297. PubMed: 10829079. 10829079 PMC18686

[21] Cherepanov PP, Wackernagel W (1995) Gene disruption in Escherichia coli: TcR and KmR cassettes with the option of Flp-catalyzed excision of the antibiotic-resistance determinant. Gene 158: 9-14. doi:10.1016/0378-1119(95)00193-A. PubMed: 7789817. 7789817

[22] Jiang M, Datta K, Walker A, Strahler J, Bagamasbad P et al. (2006) The Escherichia coli GTPase CgtAE is involved in late steps of large ribosome assembly. J Bacteriol 188: 6757-6770. doi:10.1128/JB.00444-06. PubMed: 16980477. 16980477 PMC1595513

[23] Kitagawa M, Ara T, Arifuzzaman M, Ioka-Nakamichi T, Inamoto E et al. (2005) Complete set of ORF clones of Escherichia coli ASKA library (a complete set of E. coli K-12 ORF archive): unique resources for biological research. DNA Res 12: 291-299. PubMed: 16769691. 16769691 10.1093/dnares/dsi012

[24] Eschenfeldt WH, Lucy S, Millard CS, Joachimiak A, Mark ID (2009) A family of LIC vectors for high-throughput cloning and purification of proteins. Methods Mol Biol 498: 105-115. doi:10.1007/978-1-59745-196-3_7. PubMed: 18988021. 18988021 PMC2771622

[25] Brandi A, Pietroni P, Gualerzi CO, Pon CL (1996) Post-transcriptional regulation of CspA expression in Escherichia coli. Mol Microbiol 19: 231-240. doi:10.1046/j.1365-2958.1996.362897.x. PubMed: 8825769. 8825769

[26] Lakowicz JR (2006) Principles of Fluorescence Spectroscopy: CD-ROM. Springer Verlag.

[27] Roberts RC, Toochinda C, Avedissian M, Baldini RL, Gomes SL et al. (1996) Identification of a Caulobacter crescentus operon encoding hrcA, involved in negatively regulating heat-inducible transcription, and the chaperone gene grpE. J Bacteriol 178: 1829-1841. PubMed: 8606155. 8606155 10.1128/jb.178.7.1829-1841.1996 PMC177876

[28] Niba ET, Naka Y, Nagase M, Mori H, Kitakawa M (2007) A genome-wide approach to identify the genes involved in biofilm formation in E. coli. DNA Res 14: 237-246. PubMed: 18180259. 18180259 10.1093/dnares/dsm024 PMC2779908

[29] O'Toole GA, Pratt LA, Watnick PI, Newman DK, Weaver VB et al. (1999) Genetic approaches to study of biofilms. Methods Enzymol 310: 91-109. doi:10.1016/S0076-6879(99)10008-9. PubMed: 10547784. 10547784

[30] Lederberg S, Lederberg V (1961) Hybridization between bacterial ribosomes. Exp Cell Res, 25: 198-200. PubMed: 14463415. 14463415 10.1016/0014-4827(61)90325-1

[31] Wang L, Brown SJ (2006) BindN: a web-based tool for efficient prediction of DNA and RNA binding sites in amino acid sequences. Nucleic Acids Res 34: W243-W248. doi:10.1093/nar/gkj425. PubMed: 16845003. 16845003 PMC1538853

[32] McGuffin LJ, Bryson K, Jones DT (2000) The PSIPRED protein structure prediction server. Bioinformatics 16: 404-405. doi:10.1093/bioinformatics/16.4.404. PubMed: 10869041. 10869041

[33] Ward JJ, McGuffin LJ, Bryson K, Buxton BF, Jones DT (2004) The DISOPRED server for the prediction of protein disorder. Bioinformatics 20: 2138-2139. doi:10.1093/bioinformatics/bth195. PubMed: 15044227. 15044227

[34] Young KD (2006) The Selective Value of Bacterial Shape. Microbiol Mol Biol Rev 70: 660-703. doi:10.1128/MMBR.00001-06. PubMed: 16959965. 16959965 PMC1594593

[35] Ishihama Y, Schmidt T, Rappsilber J, Mann M, Hartl FU et al. (2008) Protein abundance profiling of the Escherichia coli cytosol. BMC Genomics 9: 102. doi:10.1186/1471-2164-9-102. PubMed: 18304323. 18304323 PMC2292177

[36] Smith-Frieday MN (2009) Characterization of ProQ: An RNA Binding Protein Modulating Expression of the Osmosensor and Osmoregulator ProP of Escherichia Coli [Graduate Thesis]. Guelph: The University of Guelph . 273 p

[37] Fuentes JL, Datta K, Sullivan SM, Walker A, Maddock JR (2007) In vivo functional characterization of the Saccharomyces cerevisiae 60S biogenesis GTPase Nog1. Mol Genet Genomics 278: 105-123. doi:10.1007/s00438-007-0233-1. PubMed: 17443350. 17443350

[38] Shajani Z, Sykes MT, Williamson JR. (2011) Assembly of bacterial ribosomes. Annu Rev Biochem 80: 501-526. doi:10.1146/annurev-biochem-062608-160432. PubMed: 21529161. 21529161

[39] Koraimann G, Teferle K, Markolin G, Woger W, Högenauer G (1996) The FinOP repressor system of plasmid R1: analysis of the antisense RNA control of traJ expression and conjugative DNA transfer. Mol Microbiol 21: 811-821. doi:10.1046/j.1365-2958.1996.361401.x. PubMed: 8878043. 8878043

[40] van Biesen T, Frost LS (1994) The FinO protein of IncF plasmids binds FinP antisense RNA and its target, traJ mRNA, and promotes duplex formation. Mol Microbiol 14: 427-436. doi:10.1111/j.1365-2958.1994.tb02177.x. PubMed: 7533880. 7533880

